# 3D Correlative Imaging of Lithium Ion Concentration in a Vertically Oriented Electrode Microstructure with a Density Gradient

**DOI:** 10.1002/advs.202105723

**Published:** 2022-04-11

**Authors:** Chun Huang, Matthew D. Wilson, Kosuke Suzuki, Enzo Liotti, Thomas Connolley, Oxana V. Magdysyuk, Stephen Collins, Frederic Van Assche, Matthieu N. Boone, Matthew C. Veale, Andrew Lui, Rhian‐Mair Wheater, Chu Lun Alex Leung

**Affiliations:** ^1^ Department of Materials Imperial College London London SW7 2AZ UK; ^2^ The Faraday Institution Quad One, Becquerel Ave, Harwell Campus Didcot OX11 0RA UK; ^3^ Department of Materials University of Oxford Oxford OX1 3PH UK; ^4^ Research Complex at Harwell Rutherford Appleton Laboratory Didcot Oxfordshire OX11 0FA UK; ^5^ Department of Engineering King's College London London WC2R 2LS UK; ^6^ STFC‐UKRI Rutherford Appleton Laboratory Harwell Campus Didcot Oxfordshire OX11 0QX UK; ^7^ Faculty of Science and Technology Gunma University 1‐5‐1 Tenjin‐cho, Kiryu Gunma 376‐8515 Japan; ^8^ Diamond Light Source Harwell Science and Innovation Campus Didcot Oxfordshire OX11 0QX UK; ^9^ Radiation Physics Department of Physics and Astronomy Faculty of Sciences Ghent University Proeftuinstraat 86/N12 Gent 9000 Belgium; ^10^ Department of Mechanical Engineering University College London London WC1E 7JE UK

**Keywords:** density gradient, ion concentration, vertically oriented structure

## Abstract

The performance of Li^+^ ion batteries (LIBs) is hindered by steep Li^+^ ion concentration gradients in the electrodes. Although thick electrodes (≥300 µm) have the potential for reducing the proportion of inactive components inside LIBs and increasing battery energy density, the Li^+^ ion concentration gradient problem is exacerbated. Most understanding of Li^+^ ion diffusion in the electrodes is based on computational modeling because of the low atomic number (*Z*) of Li. There are few experimental methods to visualize Li^+^ ion concentration distribution of the electrode within a battery of typical configurations, for example, coin cells with stainless steel casing. Here, for the first time, an interrupted in situ correlative imaging technique is developed, combining novel, full‐field X‐ray Compton scattering imaging with X‐ray computed tomography that allows 3D pixel‐by‐pixel mapping of both Li^+^ stoichiometry and electrode microstructure of a LiNi_0.8_Mn_0.1_Co_0.1_O_2_ cathode to correlate the chemical and physical properties of the electrode inside a working coin cell battery. An electrode microstructure containing vertically oriented pore arrays and a density gradient is fabricated. It is shown how the designed electrode microstructure improves Li^+^ ion diffusivity, homogenizes Li^+^ ion concentration through the ultra‐thick electrode (1 mm), and improves utilization of electrode active materials.

## Graphical abstract

An interrupted in situ correlative imaging technique is developed, combining novel, full‐field X‐ray Compton scattering imaging with complementary X‐ray computed tomography that allows 3D pixel‐by‐pixel mapping of both Li^+^ stoichiometry and electrode microstructure of a LiNi_0.8_Mn_0.1_Co_0.1_O_2_ cathode to cross‐correlate the chemical and physical properties of the electrode inside a working coin cell battery.

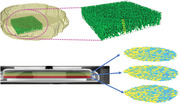

## 1 Introduction

Rechargeable Li ion batteries (LIBs) are the current battery of choice with rapidly increasing demands from electrified transport and electricity storage from intermittent renewable sources.^[^ ^1^, ^2^, ^3^ ^]^ Inside a LIB, Li^+^ ions are extracted from the cathode active material, diffused in a liquid electrolyte through the cathode porous structure to the anode, and inserted into the anode active material during charging, and the process is reversed during discharging.^[^ ^4^, ^5^, ^6^ ^]^ A key process in determining battery performance is Li^+^ ion diffusion in the pores of electrodes and insertion in the electrode active materials.^[^ ^7^, ^8^ ^]^ Theoretical studies have attributed battery capacity loss to a Li^+^ ion concentration gradient in the electrodes with a higher concentration near the separator but a lower concentration near the current collector which in turn increases the overpotential and polarization of the cell,^[^ ^9^, ^10^, ^11^, ^12^ ^]^ but the relationship between Li^+^ chemical stoichiometry distribution and electrode microstructural properties is less understood.^[^ ^13^, ^14^, ^15^ ^]^

Most battery electrodes are made by a highly productive slurry casting (SC) method which makes electrodes of 150–200 µm thickness and 20–30 vol% porosity,^[^ ^16^ ^]^ containing a random porous microstructure with highly tortuous pores that restrict Li^+^ ion diffusion through the electrode thickness,^[^ ^17^, ^18^ ^]^ which in turn causes the steep Li^+^ ion concentration gradient in the electrodes^[^ ^19^, ^20^ ^]^ and reduces the accessibility of electrode active material and battery capacity.^[^ ^21^ ^]^ Thick electrodes (≥300 µm) can reduce the proportion of inactive components (current collectors and separators) in a battery cell‐stack and increase the proportion of active components (electrodes) that contributes to energy storage,^[^ ^22^ ^]^ but thick electrodes with the conventional tortuous porous microstructure would amplify the problem of restricted Li^+^ ion diffusion and lead to under‐utilization of active materials and loss of battery capacity even with an extremely small current.^[^ ^16^, ^23^, ^24^ ^]^ Recently, there has been a growing interest in fabricating ultra‐thick electrodes (500 µm–1 mm) with vertically oriented pore channels to facilitate fast Li^+^ ion diffusion through the electrode thickness. New processing methods of making the anisotropic electrode microstructure include co‐extrusion,^[^ ^25^ ^]^ magnetic templating,^[^ ^26^ ^]^ infiltration and carbonization of natural wood,^[^ ^27^ ^]^ and directional ice templating (DIT, or freeze casting).^[^ ^28^, ^29^ ^]^ We have previously reported 900 µm thick LiCoO_2_ and LiFePO_4_ cathodes with an anisotropic microstructure made by DIT which increased gravimetric energy density by 41% at 1 C (≈1 h charge/discharge) compared with a cell‐stack of standard SC electrodes (160 µm coated on two sides of the current collector) of the same electrode material and total cell‐stack volume.^[^ ^30^, ^31^, ^32^ ^]^ However, there have been few experiments visualizing the effects of the anisotropic structure on Li^+^ ion concentration distribution to rationalize their performance improvements.

LiNi_0.8_Mn_0.1_Co_0.1_O_2_ (NMC811) is attracting considerable attention for delivering high energy density (≈capacity x average voltage) due to the Ni^2+/3+^ and/or Ni^3+/4+^ redox couples.^[^ ^33^, ^34^ ^]^ Synchrotron X‐ray computed tomography (XCT) has been used to investigate electrode physical microstructure^[^ ^35^, ^36^, ^37^, ^38^, ^39^ ^]^ although there are known X‐ray radiation induced side effects for in situ characterization of battery materials.^[^ ^40^ ^]^ As Li has one of the lowest atomic numbers (*Z*),^[^ ^41^, ^42^ ^]^ detecting the chemical composition of Li buried among high *Z* elements of cathode materials such as Ni, Mn, and Co within typical batteries such as coin cells of stainless steel casing is extremely challenging. Other in situ methods that are able to detect Li include nuclear magnetic resonance,^[^ ^43^, ^44^, ^45^ ^]^ synchrotron‐based X‐ray and neutron diffraction,^[^ ^46^, ^47^, ^48^ ^]^ and X‐ray Compton scattering (XCS).^[^ ^49^, ^50^ ^]^ Depth profile diffraction and X‐ray/neutron tomography have also been used to characterize Li concentration gradient in the electrodes.^[^ ^17^, ^19^, ^46^, ^47^, ^48^, ^51^, ^52^ ^]^ The reason for using XCS is that although the change in atoms or the total electron density of atoms caused by the change of Li^+^ stoichiometry in electrode active material is small, electron momentum of the valence electrons of the electrode active material can be obtained from XCS. The valence electrons are of particular interest as they are involved in the Li^+^ intercalation and extraction redox reaction, so calculating the change in electron momentum of the valence electrons can shed light on the change of Li^+^ stoichiometry.^[^ ^53^ ^]^ However, previous XCS experiments obtained signals through scanning an X‐ray pencil beam over the battery—one pixel after another—which was time consuming and the battery chemistry may have already changed before the scanning of the region of interest was completed.^[^ ^54^, ^55^ ^]^ Additionally, the abovementioned methods do not show electrode physical microstructure and hence, the relationship between Li chemical composition and electrode microstructure remains elusive.^[^ ^39^, ^44^, ^45^ ^]^

Here, we fabricate ultra‐thick (1 mm) NMC811 cathodes containing vertically oriented pore arrays and a density gradient through the electrode thickness with an overall porosity of 22 vol% using DIT, assembled in a standard coin cell configuration of stainless steel casing. For the first time, we develop an interrupted in situ correlative imaging technique, combining novel, full‐field X‐ray Compton scattering imaging (XCS‐I) with complementary XCT using a high energy synchrotron source X‐ray beam (115 keV) to penetrate the stainless steel battery casing which is otherwise opaque to low energy X‐ray.^[^ ^38^ ^]^ In contrast to the traditional X‐ray pencil beam, we use an X‐ray sheet beam geometry combined with a high‐energy X‐ray imaging technology (HEXITEC) detector for mapping Compton scattering energy spectra in an 80 × 80 pixels field—in a single exposure—to ensure the capture of Li^+^ ion distributions of all pixels at the same time.^[^ ^56^ ^]^ This work reports a major advancement in 3D visualizing and correlating the XCS‐I (Li^+^ chemical composition) with XCT (electrode microstructure) to rationalize that the ultra‐thick, anisotropic cathode microstructure improves Li^+^ ion diffusivity and homogenizes Li^+^ ion concentration in the electrodes. For comparison, we also fabricate and evaluate electrodes of conventional thickness and microstructure using the same materials made by standard SC. The knowledge thus gained paves the way for guiding the design of future electrode microstructure and the corresponding novel electrode manufacturing techniques to enhance battery performance.

## 2 Results and Discussion

### 2.1 Correlative High Energy Synchrotron XCS‐I and XCT

**Figure 1:**
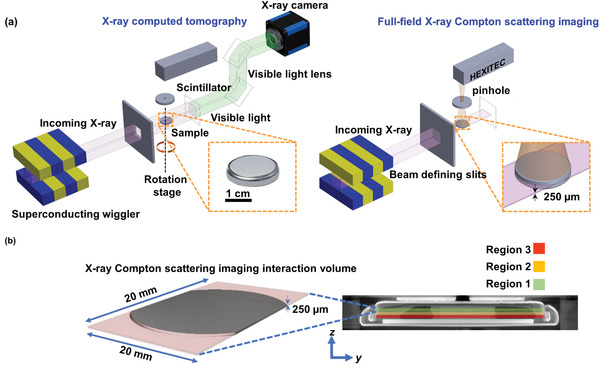
Schematics of a) experimental setup for the interrupted in situ correlative imaging technique, combining novel, full‐field X‐ray Compton scattering imaging (XCS‐I) with complementary XCT; and b) an XCS‐I interactive volume with the location of different depth regions in the cathode shown schematically on an XCT slice of the battery along the *y‐z* plane.

**Figure** **1**a shows a schematic of the experimental setup. XCT was first performed on a coin cell battery configuration where the size of the incident X‐ray beam was 25 × 5 mm^2^ (width x height) to cover the entire battery. The battery was rotated by 360° during the XCT scan, and the XCT datasets were collected by an X‐ray imaging camera. XCS‐I was then performed on the battery, with the size of the incident X‐ray beam adjusted to 25 × 0.25 mm^2^ (width x height) using beam defining slits. Since Compton scattering becomes dominant at high scattering angles for light elements such as Li,^[^ ^57^ ^]^ the scattering signals were collected by a HEXITEC detector positioned at 90° from the incident X‐ray beam, above the battery, to map the entire plane of the battery (Figure 1b). The battery position was moved vertically to collect Compton scattering signals in three depth regions through the cathode thickness with region 1 nearest the separator and region 3 nearest current collector. The XCS‐I data was then integrated to cover the same battery volume as the XCT data to provide complementary Li^+^ chemical composition and physical microstructural properties. Both the XCT and XCS‐I signals were collected after the battery was charged, and then after the same battery was discharged without opening the battery.

Previous Compton scattering synchrotron experiments used Ge‐based single photon counting detectors.^[^ ^50^ ^]^ Instead in this work, a HEXITEC detector was used, based upon a high atomic number semiconductor CdZnTe and consisting of an 80 × 80 pixels array (covering an area of 20 × 20 mm^2^), with a full energy‐resolved X‐ray spectrum measured for each pixel and all spectra captured simultaneously. Due to the higher density, larger band gap, and lower thermal charge leakage of CdZnTe than Ge, the HEXITEC detector allows measurements of X‐ray spectra to energies of 180 keV with <1 keV full width at half maximum (FWHM) energy resolution at room temperature to separate the Compton scattering signal from sharp emission lines from the detector, shielding and collimating components.^[^ ^56^ ^]^

### 2.2 Fabrication of Ultra‐Thick Electrodes Containing a Vertically Oriented Microstructure and a Density Gradient

**Figure 2:**
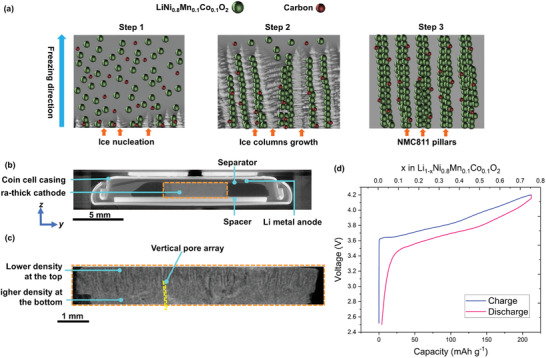
a) Schematics of the directional ice templating (DIT) process; X‐ray computed tomography (XCT) slices of b) battery containing the ultra‐thick Li_1‐_ *_x_* Ni_0.8_Mn_0.1_Co_0.1_O_2_ cathode made by DIT, and c) the magnified cathode, both along the *y‐z* plane; d) galvanostatic charge and discharge profiles of the battery in (b) at 0.5 C.

Ultra‐thick cathodes were fabricated by the DIT method.^[^ ^30^, ^31^, ^32^ ^]^ **Figure** **2**a shows a schematic of the DIT process: a vertical freezing temperature gradient was applied to an aqueous‐based slurry containing active NMC811 particles, electrically conducting carbon black nanoparticles, and a binder. Ice crystals were first rapidly nucleated at the interface of supercooling. As water continued to freeze, ice columns grew in parallel to the temperature gradient, pushing the NMC811 and carbon black particles into the regions between the ice columns. The ice crystals were then immediately sublimed, leaving aligned columns of the constituent materials. Figure S1, Supporting Information shows a schematic of the DIT apparatus. Figure S2, Supporting Information shows a scanning electron microscopy (SEM) image of the NMC811 particles (3–15 µm) from the feedstock powder. The DIT electrodes were self‐standing during the electrode formation, without substrates. Previous studies have shown no major change to the bulk structure of NMC811 in aqueous suspensions within 12 h^[^ ^58^, ^59^ ^]^ which is the case for the DIT method. Figure S3, Supporting Information shows an X‐ray diffraction pattern of the resulting DIT cathode. All the reflections in Figure S3, Supporting Information are indexed to the close‐packed oxygen lattice structure of alternating layers of Li^+^ and transition metal ions for NMC811, showing that the DIT fabrication method did not alter the material chemistry.

Figure 2b shows an XCT slice of the battery along the *y‐z* plane (the cross‐section of the battery) and the location of the ultra‐thick cathode inside the working coin cell battery that was connected to an electrochemical workstation for charge and discharge. The space around the ultra‐thick cathode was filled with liquid electrolyte. The lateral size of the electrode was controlled by the mold size used during DIT.^[^ ^60^ ^]^ Figure 2c shows a magnified XCT slice of the cathode along the *y‐z* plane where the pore phase is black and the material phase is in grey scale, showing that the electrode is ≈1 mm in thickness and the electrode microstructure contains vertically oriented pore arrays and a density gradient with a higher density in the bottom region (near the current collector that was attached to the electrode after electrode formation). This microstructure was likely due to the kinetics of ice nucleation and structure growth during the DIT process where initial rapid undercooling resulted in small ice crystals which became regions of higher electrode density at the bottom of the electrode after the ice was sublimed. Ice structures continued to grow along the temperature gradient. Due to the progressively slower heat extraction rate through the electrode thickness, the ice column diameter increased, which became regions of lower electrode density toward the top of the electrode after the ice was sublimed, resulting in a density gradient through the electrode thickness.

Figure 2d shows the galvanostatic charge and discharge profiles of the battery containing the DIT cathode at a constant rate of 0.5 C. The charge and discharge capacities were 190 and 188 mAh g^−1^ respectively, corresponding to 57 mAh cm^−2^ and 572 mAh cm^−3^ for the discharge capacity. The areal and volumetric capacities for the reported ≈100 µm thick conventional cathodes of NMC811 are 5.5–8 mAh cm^−2^ and 522–575 mAh cm^−3^, respectively.^[^ ^35^, ^61^ ^]^ For comparison, we also fabricated a cathode containing the same materials by standard SC. This electrode was made to a maximum thickness of 300 µm because it was not possible to make electrodes at >300 µm thickness using SC without cracking or delamination from the current collectors^[^ ^62^, ^63^ ^]^ as such thick SC electrodes with the conventional tortuous porous network were unable to sustain the internal strain due to the capillary force from all directions during electrode drying.^[^ ^64^, ^65^ ^]^ To further compare the electrochemical performance between the DIT and SC cathodes, Figure S4, Supporting Information shows the galvanostatic charge and discharge curves of the batteries containing the two types of cathode at increasing charge and discharge rates of 0.5, 0.75, 1, 1.5, 2 and 3 C in the potential range of 2.8–4.3 V according to standard testing methods in the literature where the actual magnitude of current density was significantly higher than the conventional electrodes due to the large electrode thickness,^[^ ^66^ ^]^ showing the gravimetric, areal and volumetric capacities. This potential range was chosen because previous studies show that the NMC811 *c* lattice parameter and crystal unit cell volume collapse rapidly above 4.3 V, leading to decreased Li^+^ mobility and difficulty in extracting more Li^+^ ions during charging.^[^ ^33^ ^]^ Figure S4, Supporting Information shows that the charge and discharge curves became distorted at fast charge and discharge rates for the SC cathode, whereas the DIT electrode still maintained adequate charge and discharge behavior, indicating higher active material utilization in the DIT electrode at increasing C rates. The overpotential was 0.20 and 0.23 V for the batteries containing the DIT and SC cathodes from 0.5 to 2 C, suggesting that the DIT electrode exhibited more favorable Li^+^ ion transport dynamics,^[^ ^67^ ^]^ The discharge capacities at increasing C rates for the two types of electrodes are shown in Figure S5, Supporting Information. Figure S6, Supporting Information shows the Coulombic efficiency of the two types of cathodes at 0.5 C over 100 cycles. Both exhibited good Coulombic efficiency reaching 99.6% and 99.3% for the DIT and SC electrodes, respectively. Figure S7, Supporting Information shows the cycling performance of the two types of cathodes at 0.5 C over 200 cycles. Both exhibited good cycling performance of 90% and 88% capacity retention for the DIT and SC electrodes respectively, consistent with the literature.^[^ ^33^ ^]^

Electrochemical impedance spectroscopy (EIS) was used to measure the overall ion diffusion coefficient *D* ^overall^ of the electrodes after galvanostatic charge and discharge. Figure S8a, Supporting Information shows the Nyquist plots of the batteries containing the DIT and SC cathodes (the region of the Nyquist plots at high frequency is magnified in Figure S8b, Supporting Information). An equivalent circuit model (Figure S9, Supporting Information) was used to represent the components involved: *R*_e_ represents the ohmic resistance of the electrode and electrolyte, *R*_CT_ represents the charge transfer resistance of the electrode, and *W* represents the Warburg impedance associated with Li^+^ ion diffusion in the electrode.^[^ ^68^ ^]^ Table S1, Supporting Information summarizes the obtained individual components and the estimated overall ion diffusion coefficient *D* ^overall^, showing *D* ^overall^ of the battery containing the DIT electrode was 6.3 × 10^−11^ cm^2^ s^−1^, 80% higher than 3.4 × 10^−11^ cm^2^ s^−1^ for the SC electrode. *D* ^overall^ of the SC electrode is comparable at ≈10^−11^ cm^2^ s^−1^ for SC electrodes of the same materials.^[^ ^23^, ^69^, ^70^ ^]^ Previous studies show that *D* ^overall^ decreased by two orders of magnitude when electrode thickness was increased from 100 to 300 µm for the SC electrodes with tortuous pores,^[^ ^23^ ^]^ whereas *D* ^overall^ of the DIT electrode was not restricted by the electrode thickness because the vertical pore arrays improved Li^+^ ion diffusion dynamics.

### 2.3 Cathode 3D Microstructure by XCT and Li^+^ Ion Diffusion Analysis

**Figure 3:**
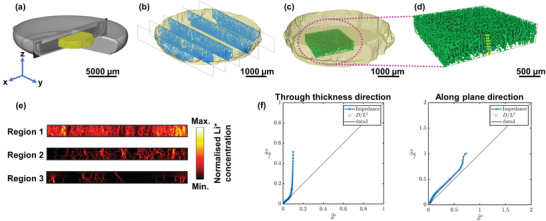
XCT results showing a) 3D reconstruction of a coin cell battery containing the DIT cathode (yellow) after one charge cycle; b) magnified 3D volume rendering of the cathode with segmented slices along the *y‐z* plane of the material (blue) and pore phases (transparent); c) 3D volume rendering of the pore phase (green) in the middle region of the DIT cathode; d) 3D enlarged pore phase showing the vertically aligned pore arrays; e) simulated Li^+^ ion flux in the *y‐z* direction in the three depth regions inside the DIT cathode; f) simulated electrochemical impedance spectroscopy (EIS) plot in the *y‐z* direction and *x*‐*y* direction of the DIT cathode. The black dotted line is the 45° slope line to show the deviation of the graph from the ideal 45° slope line. The red dot in the EIS plot indicates the position of the characteristic frequency □_c_ = *D*_e_ */L^2^* where *D*_e_ is the intrinsic Li^+^ ion diffusion coefficient in the liquid electrolyte and *L* is the length of the cuboid volume.

**Figure** **3**a shows an XCT 3D reconstruction of the coin cell containing the DIT cathode (yellow) after one charge cycle, the zoomed‐in 3D reconstruction of the cathode and the underlying segmented 2D slices along the *y‐z* plane are shown in Figure 3b, with the material phase (blue) and the pore phase that is filled with liquid electrolyte (transparent). Figure 3c shows the 3D reconstruction of the pore phase (green) which is further enlarged in Figure 3d, confirming the long‐range vertical orientation of the microstructure. The porosity ɛ was estimated from the segmented image volume at 33.3, 23.2, and 9.6 vol% in regions 1, 2, and 3 respectively, with an average porosity of 22 vol%. Figure S10, Supporting Information shows the 2D slices of the magnified DIT cathode along the *y‐z* plane using the same experimental setup but a higher resolution optical module, confirming the vertical orientation of the microstructure with a porosity gradient (pore diameters of ≤3 µm at the bottom of the electrode that gradually increased to 15–20 µm at the top of the electrode). Figure S11, Supporting Information shows the estimated volume of the DIT cathode against the distance from the current collector in the charged and discharged states, showing negligible electrode volume changes, in agreement with the small (≤5%) material volume changes of NMC811 during charge and discharge.^[^ ^71^ ^]^ Figure S11, Supporting Information shows the cathode overall volume decreases as the distance from the current collector increases. This confirms the gradient cathode structure with a higher density closest to the current collector and a lower density closest to the separator. The electrode was assembled with a lower density closest to the separator and a higher density closest to the current collector inside a battery because prior modeling studies show that a lower density at the separator/electrode interface would improve Li^+^ ion diffusion kinetics and battery electrochemical performance.^[^ ^16^, ^72^ ^]^ For comparison, Figure S12, Supporting Information shows the underlying segmented 2D slices along the *y‐z* plane and the 3D reconstruction of the SC electrode, demonstrating non‐directional pore structure. The average porosity of the SC electrode was estimated at 25%.

We simulated Li^+^ ion diffusion flux along the *x‐y* and *y‐z* planes in the porous network of the electrode based on the real electrode microstructure obtained by XCT, using an open source TauFactor platform.^[^ ^73^ ^]^ Briefly, the cathode porous network was divided into a set of discrete cuboid volumes. The Li^+^ ion diffusion flux was modeled by the solution to a system of equations (Equation (1)) which captures steady‐state diffusion problems including the fixed value (Dirichlet) conditions imposed at two parallel boundaries where Q = (0, L_x_) х (0, L_y_) х (0, L_z_) is a cuboid in 3D:

(1) $$\begin{array}{cc} \nabla^{2}\hat{C}-\frac{i\omega }{D_{e}}\hat{C}=0, & \mathrm{in}\;\Omega ,\\ \hat{C}=0, & \mathrm{on}\;T,\\ \nabla \hat{C}\cdot n=0, & \mathrm{on}\;I,\\ \hat{C}=1, & \mathrm{on}\;B, \end{array}$$

where *Ω* is the porous network inside *Q*; *T*, *I* and *B* are 2D subsets of *Q* (i.e., top, interfacial and bottom) such that ∂*Ω* = *T* ∪ *I* ∪ *B* and ∂*Ω*|_z = 0_ = *T*, ∂*Ω*|_0<z<Lz_ = *I*, ∂*Ω*|_z = Lz_ = *B*; **n** is the outward pointing unit normal to *Ω*; $\hat{C}$ is the complex Li^+^ ion concentration; *i* is the imaginary unit; *D*_e_ is the intrinsic Li^+^ ion diffusion coefficient of the liquid electrolyte, and *ω* is the frequency of the boundary simulation. The modeling uses an over‐relaxation iterative approach so the flux in each cuboid volume element depends on the flux of its face‐adjacent neighbors.^[^ ^73^ ^]^ The simulated Li^+^ ion diffusion flux in Figure 3e shows increasingly aligned Li^+^ ion diffusion in the direction through the DIT cathode thickness, the kinetically favorable direction of microscale Li^+^ ion diffusion during charge and discharge,^[^ ^74^ ^]^ from position 3 to position 1. In contrast, the simulated Li^+^ ion diffusion flux in Figure S13, Supporting Information for the SC electrode shows more flux in the direction across the electrode plane, the non‐kinetically favorable direction, as the SC process induces horizontal particle alignment due to gravity.^[^ ^21^ ^]^

Pore tortuosity *τ* quantifies the deviations of Li^+^ diffusion pathways from the straight cylindrical pores of uniform diameters (i.e., when *τ* = 1).^[^ ^75^, ^76^ ^]^ The EIS results were simulated where for each frequency, the impedance *Z* was calculated as the ratio between the amplitude of the concentration stimulus and the complex diffusion flux at the inlet boundary, and then normalized to $\tilde{Z}$.^[^ ^77^ ^]^

(2) $$\tilde{Z}=Z\frac{AD}{L}$$

where *A* and *L* are the respective total area and length of the cuboid volume normal to the direction of Li^+^ ion flux. The benefit of the simulated EIS is that Li^+^ ion diffusion along different directions and in each electrode depth region inside the electrode only can be differentiated. An idealized EIS plot of a porous electrode shows a 45° slope line in the medium frequency due to Li^+^ ion diffusion and a vertical line in the low frequency due to distributed capacitance of double layer between Li^+^ ions and the electrode material.^[^ ^78^ ^]^ Figure 3f shows the simulated EIS plot in the *y‐z* direction and in the *x*‐*y* direction in region 1 of the DIT cathode, showing a more idealized impedance response in the *y‐z* direction than in the *x*‐*y* direction. Here, the EIS data was fitted to a uniform resistor‐capacitor (RC) transmission line model (TLM, Figure S14, Supporting Information) to de‐couple the electrical resistance of the cathode solid matrix *r*_el_ and ionic resistance *r*_ion_ of the electrolyte even further down to different transport pathways and frequencies,^[^ ^79^ ^]^ and *τ* was estimated by:^[^ ^80^ ^]^

(3) $$\frac{\tau }{\varepsilon }=\frac{R_{\mathrm{ion}}A_{\mathrm{CC}}K_{0}}{L}$$

where *R*_ion_ is the sum of *r*_ion_ in TLM, *A*_cc_ is the macroscopic current collector area and *K*_0_ is the electrical conductivity of the active material. The directional Li^+^ ion diffusion coefficient *D* ^directional^ in the porous network of the electrode was estimated by:^[^ ^81^ ^]^

(4) $$D^{\mathrm{directional}}=D\frac{\varepsilon }{\tau }$$

*τ* in the *y‐z* direction was 1.1, 2.3, and 2.8 in regions 1, 2, and 3 for the DIT electrode, which are significantly lower than *τ* in the *x‐y* direction in the same regions (8.7, 10.5, and 11.2). In contrast, *τ* in the *y‐z* direction was 11.1, 12.3, and 14.8 in regions 1, 2, and 3 for the SC electrode, higher than *τ* in the *x‐y* direction in the same regions (9.6, 10.5, and 11.3). Here, the DIT electrode achieved a significantly lower *τ* in the *y‐z* direction even with a thickness being greater than three times larger than the SC electrode. Our results revealed an increased *D* ^directional^ in the porous network of the DIT cathode from 4.8 × 10^−9^ cm^2^ s^−1^ (region 3) to 4.2 × 10^−8^ cm^2^ s^−1^ (region 1) in the *y‐z* direction, which is also an order of magnitude higher than the corresponding *D* ^directional^ in the same regions in the *x‐y* direction. **Table** **1** summarizes the estimated ɛ, *τ* and *D* ^directional^.

**Table 1:** A summary of porosity *ɛ*, pore tortuosity *τ* and directional Li^+^ ion diffusion coefficient in the pore network *D* ^directional^ in three depth regions of the ultra‐thick Li_1‐_ *_x_* Ni_0.8_Mn_0.1_Co_0.1_O_2_ cathode made by directional ice templating (DIT). *τ* and *D* ^directional^ were estimated in the *x‐y* direction (along the cathode plane) and in the *y‐z* direction (through the cathode thickness)

Depth region	Porosity *ɛ*	Pore tortuosity *τ*		Directional Li^+^ ion diffusion coefficient in the pore network *D* ^directional^	
	vol%	a.u.		x 10^−9^ cm^2^ s^−1^	
		*x‐y* direction	*y‐z* direction	*x‐y* direction	*y‐z* direction
1	33.3	8.7	1.1	5.3	42.1
2	23.2	10.5	2.3	3.1	14.0
3	9.6	11.2	2.8	1.2	4.8

### 2.4 3D Spatial Distribution of Li^+^ Chemical Composition in the Cathode by XCS‐I and XCT

To obtain 3D spatially resolved Li^+^ chemical composition and correlate with the cathode physical microstructural properties, we used the binarized XCT cathode image volume to extract the XCS‐I results in each depth region, so the obtained Compton scattering energy spectra corresponded to the cathode volume only. The summation of XCS energy spectra among all pixels in the three depth regions of the DIT cathode in the charged and discharged states are shown in Figures S15a–c and S16a–c, Supporting Information, respectively. The peak at 93.9 keV is from the X‐ray inelastically scattered from the cathode which mainly consists of the NMC811 material^[^ ^82^ ^]^ The other small peaks are due to the fluorescent X‐rays of K_*α*_ and K_*β*_ lines for Cd (22.9 and 26.1 keV) and Te (27.2 and 31.0 keV) from the detector,^[^ ^83^ ^]^ a mixture of background and fluorescent X‐rays (50 – 85 keV) of W (pin‐hole) and Pb (shielding of X‐rays),^[^ ^83^ ^]^ and the low energy threshold of the detector (≈5 keV).^[^ ^84^ ^]^ The intensity of the peak *dN* is related to the electron density of the NMC811 material *ρ*_e_ through:^[^ ^50^, ^85^ ^]^

(5) $$dN=\varphi_{0}t_{1}t_{2}\rho_{e}dV\frac{d\sigma_{KN}}{d\Omega }$$

where *φ*_0_ is the photon flux of the incident X‐ray, *t*_1_ is the incident X‐ray transmittance from the entrance surface to the probing volume of the cathode, *t*_2_ is the scattered X‐ray transmittance from the probing volume to the exit surface, *dV* is the probing volume, and *dσ_KN_*/*dΩ* is the Klein‐Nishina differential cross section.

**Figure 4:**
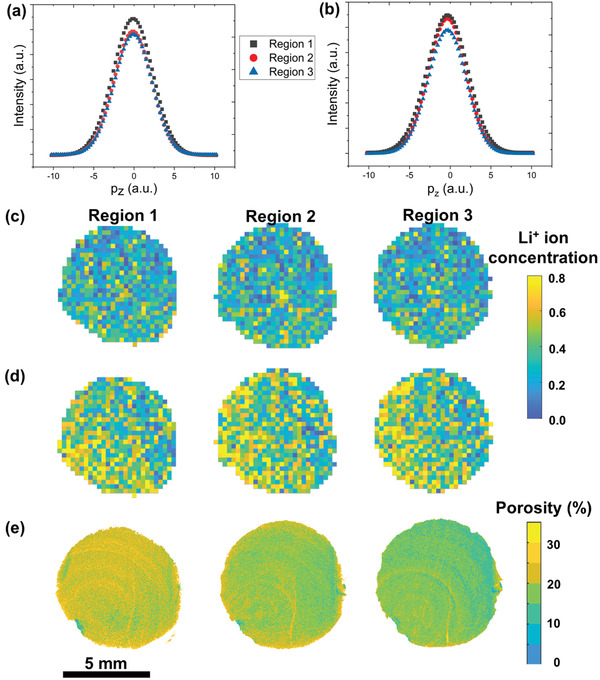
XCS‐I results showing electron momentum *p_z_* profile in three depth regions of the cathode in the a) charged, and b) discharged states and maps of the lateral distribution of Li^+^ stoichiometry pixel‐by‐pixel in the three depth regions inside the cathode in the c) charged and d) discharged states. XCT results showing e) lateral distribution of electrode porosity in the three depth regions inside the cathode. During all the above characterization, the cathode remained inside the battery.

A Compton profile was generated at each pixel from the Compton scattering energy peak at 93.9 keV through:^[^ ^86^ ^]^

(6) $$\frac{p_{z}}{mc}≅\frac{E_{2}-E_{1}+\left(\frac{E_{2}E_{1}}{mc^{2}}\right)\left(1-\cos\theta \right)}{\sqrt{E_{1}^{2}+E_{2}^{2}-2E_{1}E_{2}\cos\theta }}$$

where *p_z_* is a projection of the electron momentum of electrons in both core and valence orbitals of the Li_1‐_ *_x_* Ni_0.8_Mn_0.1_Co_0.1_O_2_ molecule, *E*_1_ and *E*_2_ are energies of the incident and Compton scattered X‐rays respectively, *m* is the electron mass, *c* is the speed of light and *θ* is the scattering angle. **Figure** **4**a,b shows the summation of the Compton profile among all pixels in the three depth regions of the cathode in the charged and discharged states. The valence electron momentum (−1 < *p_z_* < 1, low kinetic energy range for the slowly moving valence electrons) was extracted from the core electron momentum (*p_z_* < −5 and *p_z_* > 5, the high kinetic energy range). These energy ranges were chosen from the difference in the Compton scattering energy spectra between the fully charged and discharged states of the cathode from previous studies.^[^ ^50^, ^54^ ^]^ We then estimated the proportion of occupied valence electron orbitals among the core electron orbitals in the Li_1‐_ *_x_* Ni_0.8_Mn_0.1_Co_0.1_O_2_ molecule through an *S‐*parameter:^[^ ^50^ ^]^

(7) $$S=\frac{S_{L}}{S_{H}}$$

where *S*_L_ and *S*_H_ are the integral of low and high electron momentum densities in the electron momentum profiles, as shown below:^[^ ^87^ ^]^

(8) $$S_{L}=\int_{-1}^{1}J\left(p_{z}\right)dp_{z}$$

(9) $$S_{H}=\int_{-5}^{-1}J\left(p_{z}\right)dp_{z}+\int_{1}^{5}J\left(p_{z}\right)dp_{z}$$

We quantified Li^+^ stoichiometry in Li_1‐_ *_x_* Ni_0.8_Mn_0.1_Co_0.1_O_2_ through finding the relationship between the *S‐*parameter and “1‐*x*.” The theoretical Compton profiles of the core and valence electrons in Li_1‐_ *_x_* Ni_0.8_Mn_0.1_Co_0.1_O_2_ for x = 1, 0.75, 0.5, 0.25, and 0 were computed using first‐principles Korringa‐Kohn‐Rostoker coherent‐potential‐approximation (KKR CPA) calculations within the framework of local spin‐density approximation^[^ ^88^ ^]^ and Mann's numerical relativistic Hartree‐Fock wavefunction model.^[^ ^89^, ^90^ ^]^ We calculated the *S‐*parameter from the theoretical Compton profiles and performed inductively coupled plasma analysis on the post‐mortem cathode after the charge and discharge cycles to calibrate the calculated *S‐*parameter with the experimental results. Figure S17, Supporting Information shows the *S‐*parameter varies linearly with the chemical stoichiometry *“*1‐*x”* in Li_1‐_ *_x_* Ni_0.8_Mn_0.1_Co_0.1_O_2_ which is directly proportional to the Li^+^ ion concentration.

Figure 4c,d shows mapping of the lateral distribution of Li^+^ chemical stoichiometry pixel‐by‐pixel in the three depth regions of the cathode in the charged and discharged states inside the working battery. The Li^+^ stoichiometry was higher in all the three depth regions in the discharged state than in the charged state because Li^+^ ions were removed out of Li_1‐_ *_x_* Ni_0.8_Mn_0.1_Co_0.1_O_2_ in the charged state and intercalated back into Li_1‐_ *_x_* Ni_0.8_Mn_0.1_Co_0.1_O_2_ in the discharged state. The lateral variations in Li^+^ stoichiometry within each depth region in Figure 4c,d from the XCS‐I results correlate microstructural changes from the XCT results, for example, the porosity mapping within each depth region in Figure 4e. The higher Li^+^ stoichiometry on the left part of the cathode in Figure 4d corroborates the slightly higher porosity on the left part of the cathode in Figure 4e. The slight lateral porosity inhomogeneity may be due to an inhomogeneous undercooling interface during the DIT fabrication process as ice nucleation was first initiated at the undercooling interface followed by vertical ice structure growth. This newly developed insight supports the understanding of electrode internal microstructure formation not only vertically, but also laterally, which subsequently influences Li^+^ chemical composition distribution in the electrode.

**Table** **2** summarizes the *S‐*parameter and Li^+^ stoichiometry (“1‐*x*”) in Li_1‐_ *_x_* Ni_0.8_Mn_0.1_Co_0.1_O_2_ among all pixels in each depth region of the cathode in the charged and discharged states. The results were cross‐correlated to the electrode physical microstructural properties in Table 1 and rationalized the highest Li^+^ stoichiometry in region 1 due to the highest *ɛ*, lowest *τ*, and highest *D* ^directional^ in the *y‐z* direction, providing experimental evidence of microstructural influences on 3D Li^+^ ion concentration distributions. Previous studies reported thick electrodes (350 – 500 µm) with non‐directional microstructure typically exhibit a 25–100% Li^+^ ion concentration gradient from the region nearest to the separator to the region nearest to the current collector.^[^ ^91^, ^92^ ^]^ Here, the Li^+^ ion concentration gradient was reduced by only 3% from position 1 to 2, and by 16% from position 2 to 3 through the ultra‐thick cathode (1 mm), demonstrating that the cathode microstructure of vertical pore arrays with a density gradient played a vital role in homogenizing Li^+^ ion concentration and improving active material utilization while maintaining a relatively low overall porosity.

**Table 2:** A summary of the *S‐*parameter and Li^+^ stoichiometry (*“*1‐*x”*) in Li_1‐_ *_x_* Ni_0.8_Mn_0.1_Co_0.1_O_2_ in three depth regions of the cathode inside a working battery in the charged and discharged states

State	Depth region	S‐parameter	Li^+^ stoichiometry (“1‐*x*”) in Li_1‐_ *_x_* Ni_0.8_Mn_0.1_Co_0.1_O_2_
		a.u.	a.u.
Charged	1	0.88	0.10
	2	0.72	0.03
	3	0.65	0.00
Discharged	1	2.40	0.76
	2	2.34	0.73
	3	2.06	0.61

## 3 Conclusions

An ultra‐thick (1 mm) Li_1‐_ *_x_* Ni_0.8_Mn_0.1_Co_0.1_O_2_ (NMC811) cathode containing an anisotropic microstructure of vertically aligned pore arrays and a density gradient was fabricated by a DIT technique. Electrochemical evaluation shows that the DIT electrode exhibited faster Li^+^ ion diffusion, increased capacity, and rate capability than the conventional electrode of non‐directional microstructure made by standard SC, despite the DIT electrode being greater than three times thicker than the SC electrode where thick electrodes have the potential for reducing the proportion of inactive components in battery cell stacks and increase energy density at the device level. Although it is usually challenging to image light elements such as Li, here, we pioneer an interrupted in situ correlative imaging technique, combining novel, full‐field XCS‐I with complementary XCT that allows 3D pixel‐by‐pixel mapping and correlation between Li^+^ chemical stoichiometry and electrode physical microstructure inside a working coin cell battery. The results show that the Li^+^ ion concentration gradient inside the ultra‐thick cathode from the separator to the current collector was significantly alleviated while maintaining an overall porosity of 22 vol%, rationalizing the improved electrochemical performance. These results demonstrate that our approach to manipulate electrode microstructure improves Li^+^ ion diffusion and electrode active material utilization in LIBs.

## 4 Experimental Section

### Electrode Fabrication and Electrochemical Testing

NMC811 powder was provided by Targray, UK. An electrode slurry was prepared by homogeneously mixing NMC811, Super P electrical conductivity enhancer, and binder at a weight ratio of 90: 5: 5. For fabricating the electrodes by DIT, the slurry was directionally frozen in a custom‐made 3D printed acrylonitrile butadiene styrene (ABS) mold on a copper cold finger, one end of which was immersed in liquid nitrogen. Freezing involved cooling from room temperature to below 0 °C until fully solid. The temperature was measured by a thermocouple inserted in the copper cold finger. The free‐standing frozen electrodes were extracted from the molds and freeze‐dried. The cathodes were assembled into standard stainless steel coin cells (CR2032) in an Ar filled glovebox with Li foil counter electrode, polyethylene separator, and electrolyte of 1 m LiPF_6_ dissolved in a mixed solvent of ethylene carbonate (EC) and diethyl carbonate (DEC) in 1:1 v/v. Electrolyte chemistry containing other additives such as 1 vol% tris(trimethylsilyl)phosphite (TMSPi) in combination with 1 vol% vinylene carbonate (VC) or higher salt concentrations have been reported to increase capacity retention after cycling^[^ ^93^, ^94^ ^]^ and may increase active material utilization using the novel electrode microstructure through optimizing the proportion of different solvents in the electrolyte to optimize viscosity and electrochemical properties.^[^ ^95^ ^]^ The cells were galvanostatically charged and discharged using a Gamry Reference 600/EIS300 potentiostat/galvanostat.

### XCT

Imaging was conducted using a transmission geometry bent Laue double crystal monochromator with a 115 keV X‐ray beam at the beamline I12‐JEEP (Joint Engineering, Environmental and Processing), Diamond Light Source.^[^ ^96^ ^]^ Radiation damage in solids with such high energy photon beams was known to be negligible compared with low energy photon beams (<10 keV) because such high‐energy photon beams can penetrate through matter,^[^ ^97^ ^]^ for example, the mass absorption coefficients of Ni, Mn and Co was reduced exponentially from 10^4^ cm^2^ g^−1^ at 1 keV to <0.1 cm^2^ g^−1^ at 115 keV.^[^ ^97^ ^]^

A synchrotron XCT scan was performed at the pristine state of the coin cell, after the cell was fully charged and after fully discharged. The incident X‐ray beam with a size of 25 × 5 mm^2^ probed the entire battery. Each tomogram was captured by a PCO.Edge 5.5 sCMOS camera coupled with I12's optical module 2 and 0.3 mm thick single crystal LuAG:Ce; the optics allowed a field of view of 20 mm × 10 mm, and a pixel size of 7.91 µm. I12's optical module 3 allowed a field of view of 8 mm × 7 mm, and a pixel size of 3.24 µm. A double field of view measurement technique was used for the XCT measurements with off‐centered samples because the sample horizontal size exceeded a single field of view.^[^ ^98^ ^]^ The XCT scan consisted of 3600 projections over 360° with an exposure time of 9 ms per projection. The detailed setup is shown in Figure 2a. Tomographic reconstruction was performed using the SAVU system.^[^ ^99^ ^]^ A filtered back projection algorithm was used,^[^ ^100^ ^]^ as implemented in the ASTRA toolbox,^[^ ^101^ ^]^ and ring artifact removing algorithm was applied.^[^ ^102^ ^]^ Lab source XCT was performed on a Zeiss Xradia for the SC electrodes due to the small thickness of the electrode. The resulting scans were reconstructed into a 3D volume using filtered back projection and beam hardening correction algorithms embedded in a Scout‐and‐Scan Control System Reconstructor (Zeiss).

### Signal Processing for XCT

3D image processing, quantification, and data visualization were performed using a combination of MATLAB 2019b, ImageJ, and Avizo 2019.2. To enhance the signal‐to‐noise ratio, the electrode structure was first extracted as a mask by performing a series of processing steps on the raw XCT image volumes, including a 3D median filter with a kernel of 3 × 3 × 3, morphological opening with a radius of 11 voxels, interactive thresholding, and component analysis was then connected to extract the electrode. After that, unsharp masking was used to deblur the image volume and the electrode was then segmented as a binary image.^[^ ^32^, ^103^ ^]^ Porosity of the cathode per region was calculated by dividing the segmented pore volume by the filled volume of the cathode.

### XCS‐I

The coin cell battery with a beam size of 25 × 0.25 mm^2^ was probed and the XCS signals were collected through a 2 mm thick W plate with a 0.2 mm pinhole at a distance of 160 mm perpendicular to the incident X‐ray beam and parallel to the flat side of the coin cell battery using a 2D high energy X‐ray imaging technology (HEXITEC) CdZnTe detector (2 mm thick),^[^ ^56^, ^104^ ^]^ a further 150 mm was positioned from the pinhole, see Figure 2a. Pb shielding was used to shield the detector from non‐Compton scattering X‐rays. The HEXITEC detector had 80 × 80 pixels on a 0.25 mm pitch. Given the pinhole geometry, each pixel in the image corresponded to 0.27 × 0.27 mm^2^ at the sample. The detector was read out using the SpecXiDAQ software,^[^ ^105^ ^]^ operating at a continuous frame rate of 9 kHz with the energy and position of every individual X‐ray photon output as raw data. The recording of the data from the camera began when the data acquisition software received a trigger,^[^ ^105^ ^]^ taking *≈*12 min per region and 36 min in total to complete. The raw data were converted into a spectrum per pixel by software.^[^ ^105^ ^]^ Charge sharing events that spanned more than 2 ing pixels were excluded from the spectral reconstructions. The HEXITEC detector collects X‐ray spectra up to 180 keV at room temperature.^[^ ^56^ ^]^ The average energy resolution (FWHM) of a pixel was measured to be 0.79 ± 0.15 keV using the 59.54 keV line from an Am‐241 sealed source.^[^ ^105^ ^]^ The temperature of the CdZnTe detector was maintained at 18 °C with a bias voltage of −1200 V applied. It was focused on analyzing the peak at 93.9 keV of the XCS energy spectra through peak fitting using the Lorentz Gaussian function. The battery cell was moved to 3 different positions through the cathode thickness while collecting Compton scattering signals. Correction factors of 1.015,1.010 and 1.005 were applied to correct for sample attenuation variations. Images of the Compton signal were formed by integrating the counts around 93 keV with the scanning duration.

### Correlating XCT with XCS‐I

For each coin cell, the electrode structure was evenly divided into 3 image segments, separated along the *y‐z* direction, which corresponded to the 3 depth regions at which the XCS‐I signals were collected. The 3D XCT volume was converted into three 2D images. These 3 image volumes were downsampled to match the field of view and voxel resolution of the Compton scattering image. This was followed by image registration, and then image multiplications, resulting in XCT images overlaid with XCS‐I results.

### Statistical Analysis

Image processing was carried out using MATLAB 2019b, ImageJ, and Avizo 2019.2. Data processing was operated by the Origin software (OriginLab) and MATLAB 2019b. Tomographic reconstruction was performed using several python packages, including SAVU,^[^ ^99^ ^]^ a filtered back projection algorithm^[^ ^100^ ^]^ in the ASTRA toolbox,^[^ ^101^ ^]^ and ring artifact removing algorithm.^[^ ^102^ ^]^ Lab source XCT scans were reconstructed into a 3D volume using filtered back projection and beam hardening correction algorithms embedded in a Scout‐and‐Scan Control System Reconstructor (Zeiss).

## Conflict of Interest

The authors declare no conflict of interest.

## Author Contributions

C.H., M.D.W., and T.C. conceived the idea and designed the experiments. C.H. conducted the experiments of electrode fabrication, battery assembly, and electrochemical tests. C.H., M.D.W., K.S., E.L., T.C., O.V.M., S.C., F.V.A., M.N.B., M.C.V., and A.L. conducted the beamtime experiments, improved the technique, and obtained results. C.H., M.D.W., K.S., E.L., and C.L.A.L. analyzed data. C.H., M.D.W., O.V.M., and C.L.A.L. wrote the manuscript. All authors discussed the results and commented on the manuscript.

## Supporting information

Supporting Information

## Data Availability

The data that support the findings of this study are available in the supplementary material of this article.
