# Supplementary material for: 3D Correlative Imaging of Lithium Ion Concentration in a Vertically Oriented Electrode Microstructure with a Density Gradient
Source: Adv Sci (Weinh). 2022 Apr 11;9(16):2105723. doi: 10.1002/advs.202105723 (PMC9165496; doi:10.1002/advs.202105723)
Supplement: Supplementary file 1 — Supporting Information [file ADVS-9-2105723-s001.pdf]

Supporting Information

Three-dimensional correlative imaging of lithium ion concentration in a vertically oriented electrode microstructure with a density gradient

Chun Huang[a,b,c,d,e]*, Matthew D. Wilson[f], Kosuke Suzuki[g], Enzo Liotti[c], Thomas Connolley[h], Oxana V. Magdysyuk[h], Stephen Collins[h], Frederic Van Assche[i], Matthieu N. Boone[i], Matthew C. Veale[f], Andrew Lui[c], Rhian-Mair Wheater[f] and Chu Lun Alex Leung[j,d]

[a] *Department of Materials, Imperial College London, London, SW7 2AZ, UK*

[b] *The Faraday Institution, Quad One, Becquerel Ave, Harwell Campus, Didcot, OX11 0RA, UK*

[c] *Department of Materials, University of Oxford, Oxford, OX1 3PH, UK*

[d] *Research Complex at Harwell, Rutherford Appleton Laboratory, Didcot, Oxfordshire, OX11 0FA, UK*

[e] *Department of Engineering, King's College London, London, WC2R 2LS, UK*

[f] *STFC-UKRI, Rutherford Appleton Laboratory, Harwell Campus, Didcot, Oxfordshire, OX11 0QX, UK*

[g] *Faculty of Science and Technology, Gunma University, 1-5-1 Tenjin-cho, Kiryu, Gunma, 376-8515, Japan*

[h] *Diamond Light Source, Harwell Science and Innovation Campus, Didcot, Oxfordshire, OX11 0QX, UK*

[i] *Radiation Physics, Department of Physics and Astronomy, Faculty of Sciences, Ghent University; Proeftuinstraat 86/N12, 9000 Gent, Belgium*

[j] *Department of Mechanical Engineering, University College London, London, WC1E 7JE, UK*

*Corresponding author's email address: a.huang@imperial.ac.uk

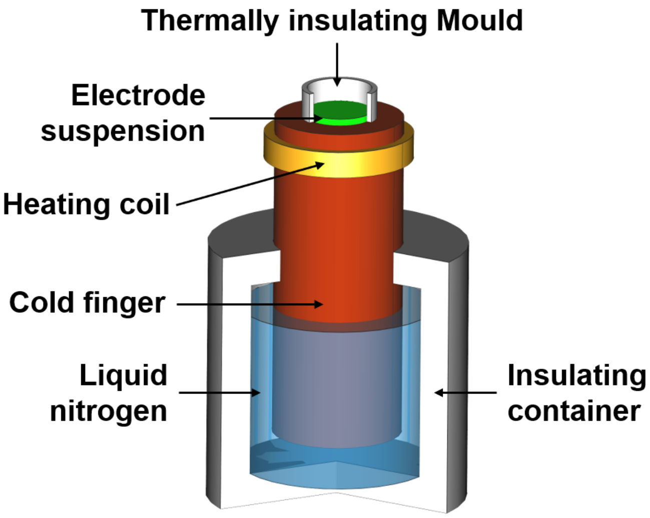

Fig. S1: Schematic of the directional ice templating (DIT) apparatus.

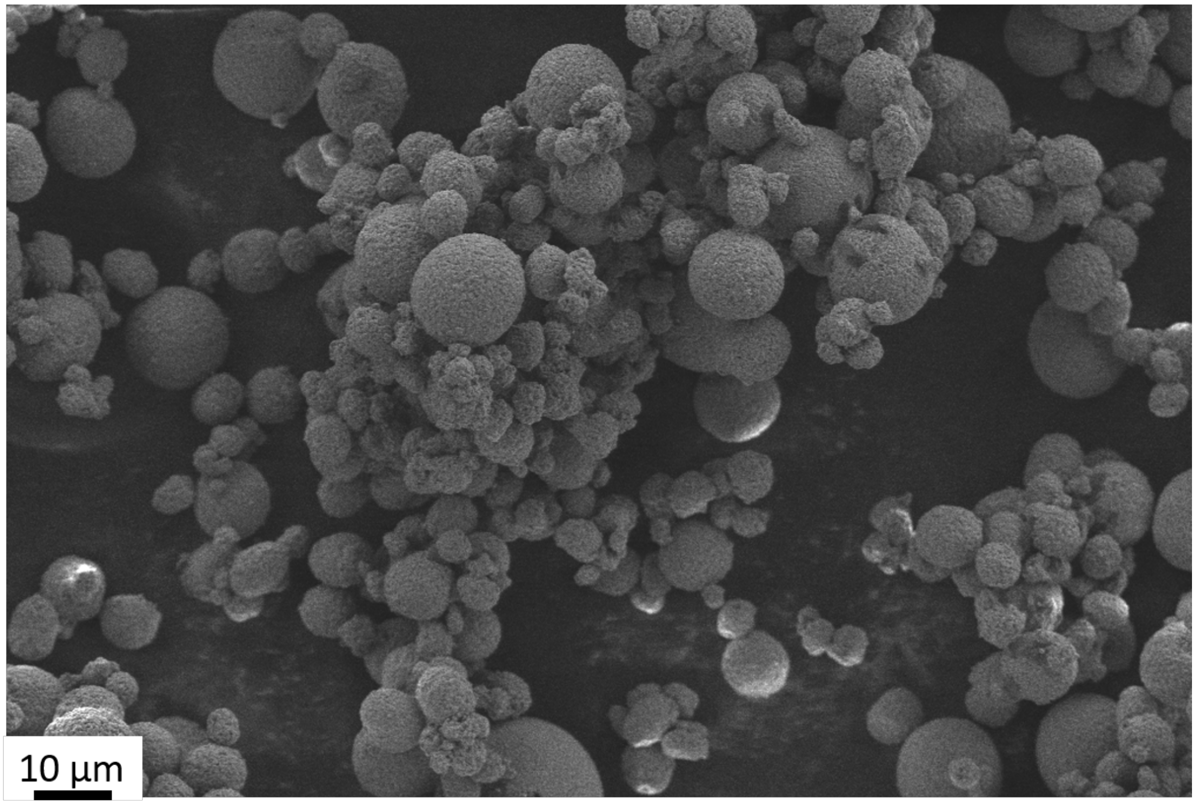

Fig. S2: SEM image of NMC811 feedstock powder particles.

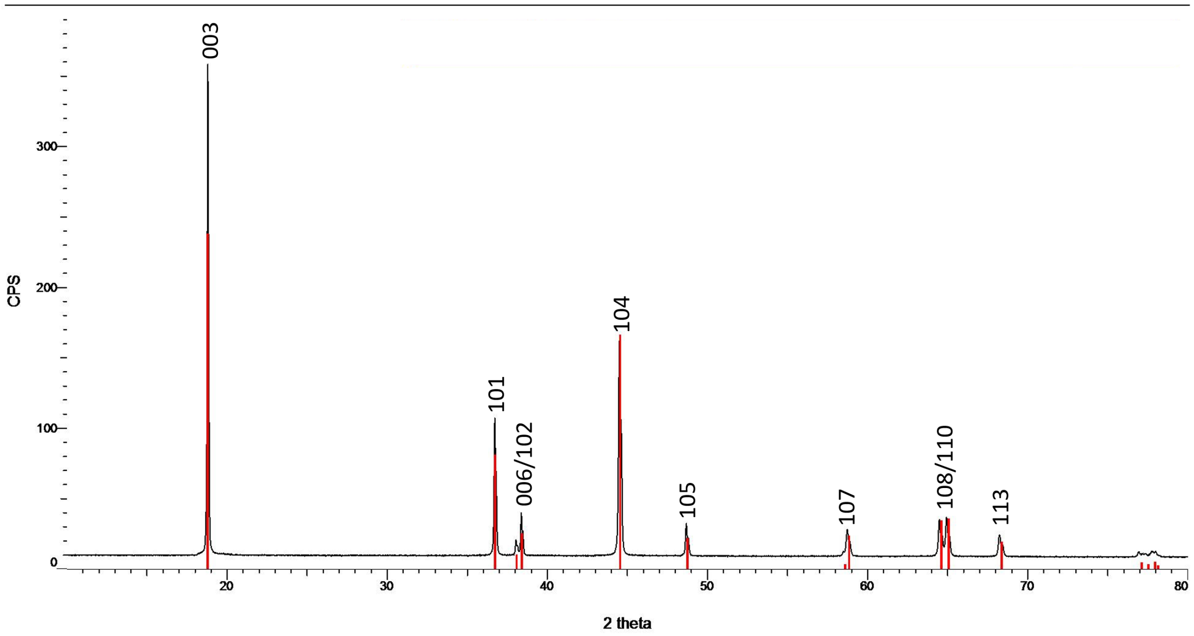

Fig. S3: XRD pattern of the as-fabricated NMC811 cathode by DIT.

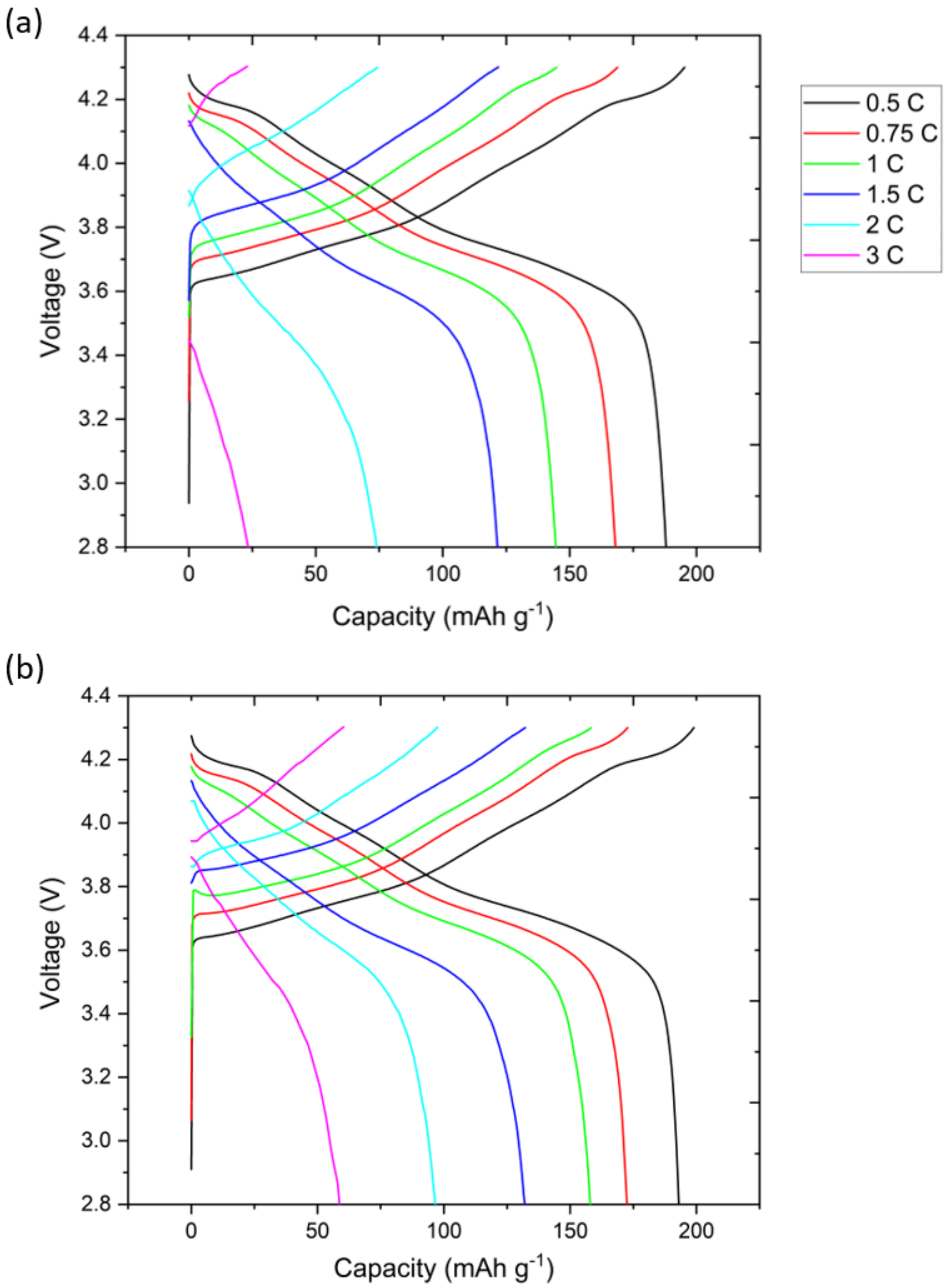

Fig. S4: Galvanostatic charge and discharge curves of coin cell batteries containing (a) conventional NMC811 cathode made by the standard slurry casting (SC) method; and (b) NMC811 cathode made by the DIT method.

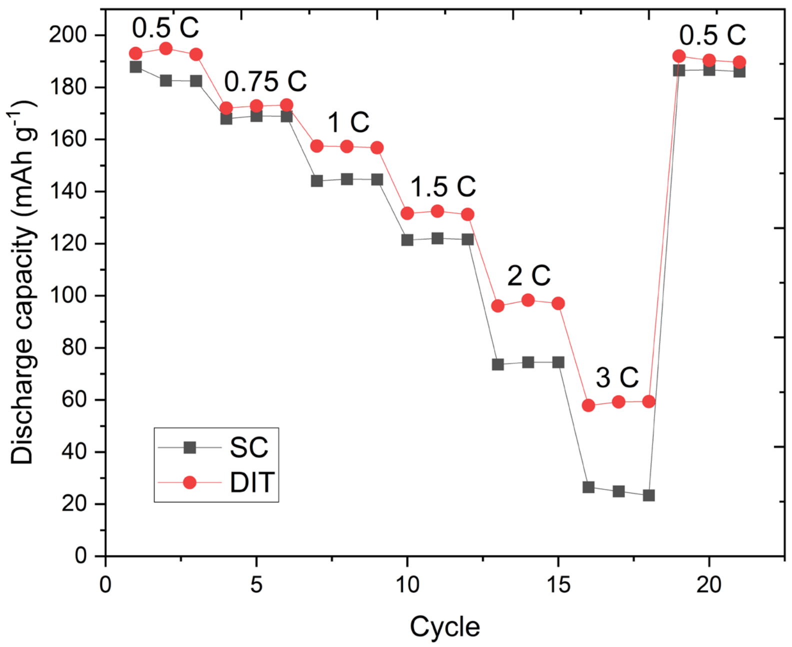

Fig. S5: Rate capability of the coin cell batteries containing conventional NMC811 cathode made by the standard SC method and NMC811 cathode made by the DIT method.

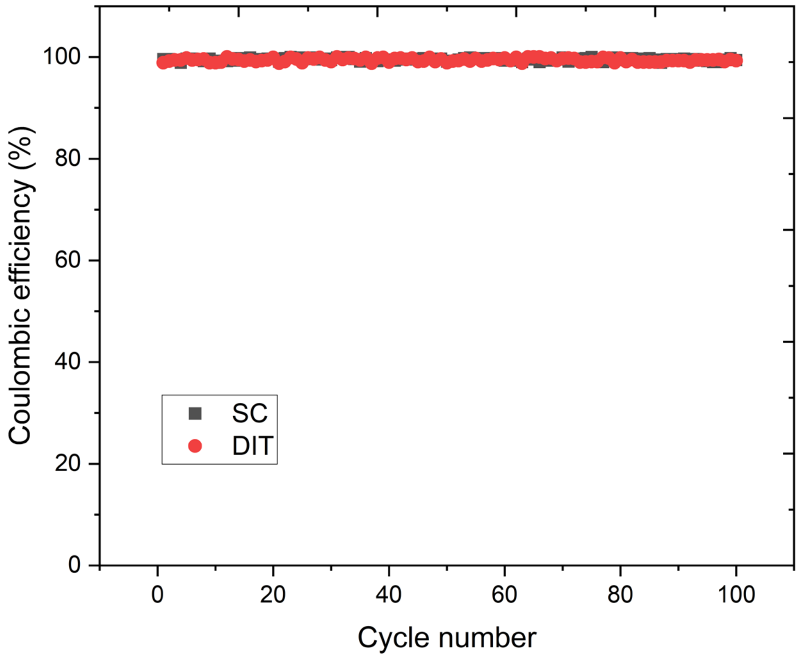

Fig. S6: Coloumbic efficiency of the coin cell batteries containing conventional NMC811 cathode made by the standard SC method and NMC811 cathode made by the DIT method at 0.5 C.

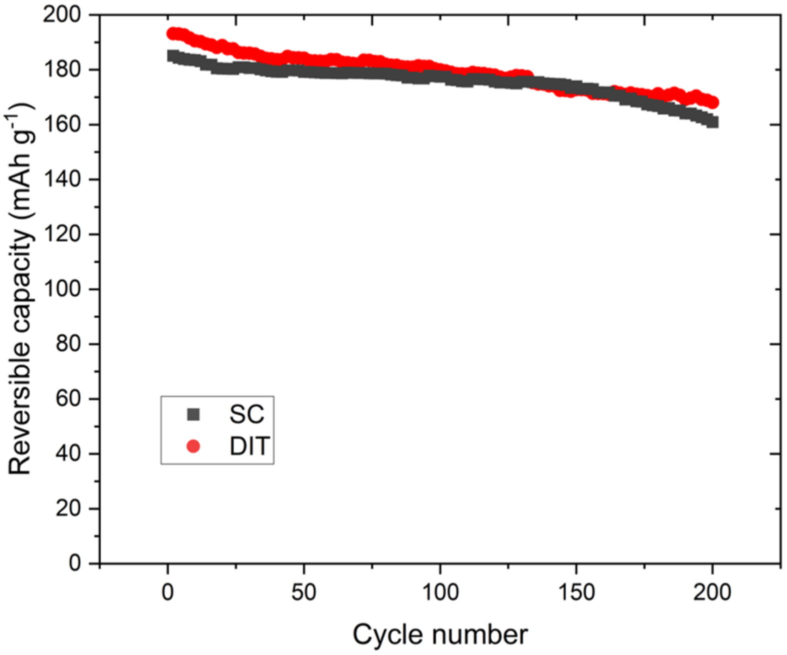

Fig. S7: Cycling performance of the coin cell batteries containing conventional NMC811 cathode made by the standard SC method and NMC811 cathode made by the DIT method at 0.5 C.

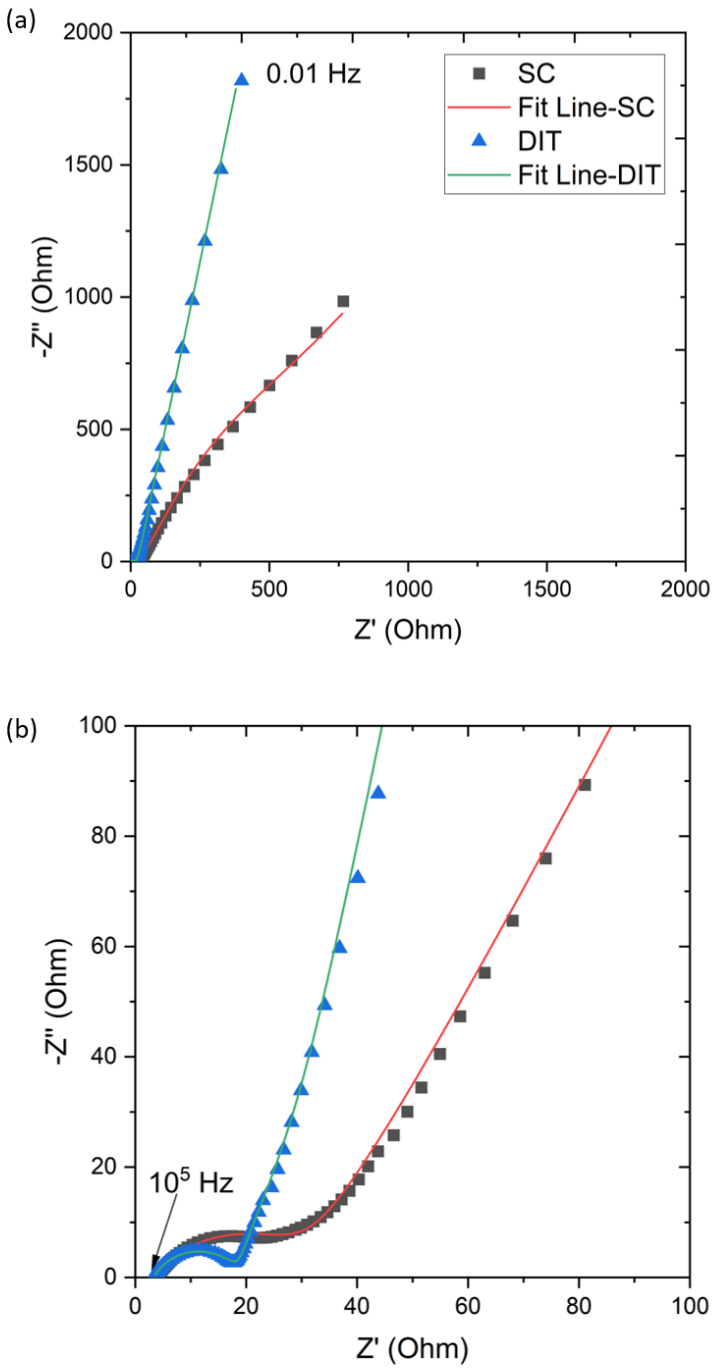

Fig. S8: (a) Nyquist plot of batteries containing the DIT cathode and the conventional cathode made by SC at open circuit voltage at 10^5 – 0.01 Hz where the high frequency region of the Nyquist plot is magnified in (b).

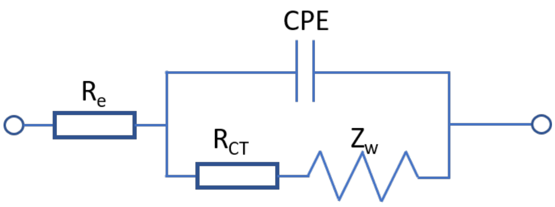

Fig. S9: Equivalent circuit model for fitting the results measured by EIS.

Method for estimating the overall Li^+ ion diffusion coefficient $D^{overall}$ from the EIS results:

The *W* component models semi-infinite linear diffusion with a straight line at a phase of 45° according to:

$$Z_w = \sigma\omega^{-1/2} - j\sigma\omega^{-1/2} \quad (1)$$

where *σ* is the Warburg coefficient, and *ω* is the frequency. The *CPE* gives a capacitor–like straight line at a phase of (90×n)° according to:

$$Z_Q = \frac{1}{Q_0(j\omega)^n} \quad (2)$$

where *n* is the constant phase and Q_0 is the constant phase coefficient. Using a *CPE* element with n fixed at ½, equations (1) and (2) are related by:

$$\sigma = \frac{1}{Q_0\sqrt{2}} \quad (3)$$

The Li^+ ion diffusion coefficient $D^{overall}$ was calculated according to:

$$D^{overall} = \frac{R^2T^2}{2A^2n^4F^4C^2\sigma^2} \quad (4)$$

where *R* is gas constant (8.314 J mol^{-1} K^{-1}), *T* is the absolute temperature (K), *A* is the electrode area (cm^2), *n* is the number of electrons involved in the redox process, *C* is the shuttle concentration (mol cm^{-3}) and *F* is the Faraday constant (96,486 C mol^{-1}) [S1]. Table S1 shows the key parameters from the equivalent circuit model and the Li^+ ion diffusion coefficient *D* for the batteries containing the DIT cathode and the conventional cathode made by SC. Since the fundamental of the fitting is based on the least square principle to acquire the minimum value of the least square coefficient between the fitting data and the experimental data [S2], Table S1 also shows the Chi-square values (χ^2) calculated from the equivalent circuit models where the small χ^2 values indicate good matching with the experimental data and estimated parameters from the equivalent circuit model.

Table 1: Electrode key components obtained from equivalent circuit model generated from the EIS results and the estimated Li^+ ion diffusion coefficient *D* for the batteries containing the cathode made by DIT and the conventional cathode made by SC

Electrode	R_e (Ω)	R_{CT} (Ω)	Q_0 (S s^a)	D (cm^2 s^{-1})	χ^2
SC	3.6	26.7	3.27×10^{-2}	3.4×10^{-11}	4.87×10^{-4}
DIT	3.1	17.4	4.45×10^{-2}	6.3×10^{-11}	1.27×10^{-4}

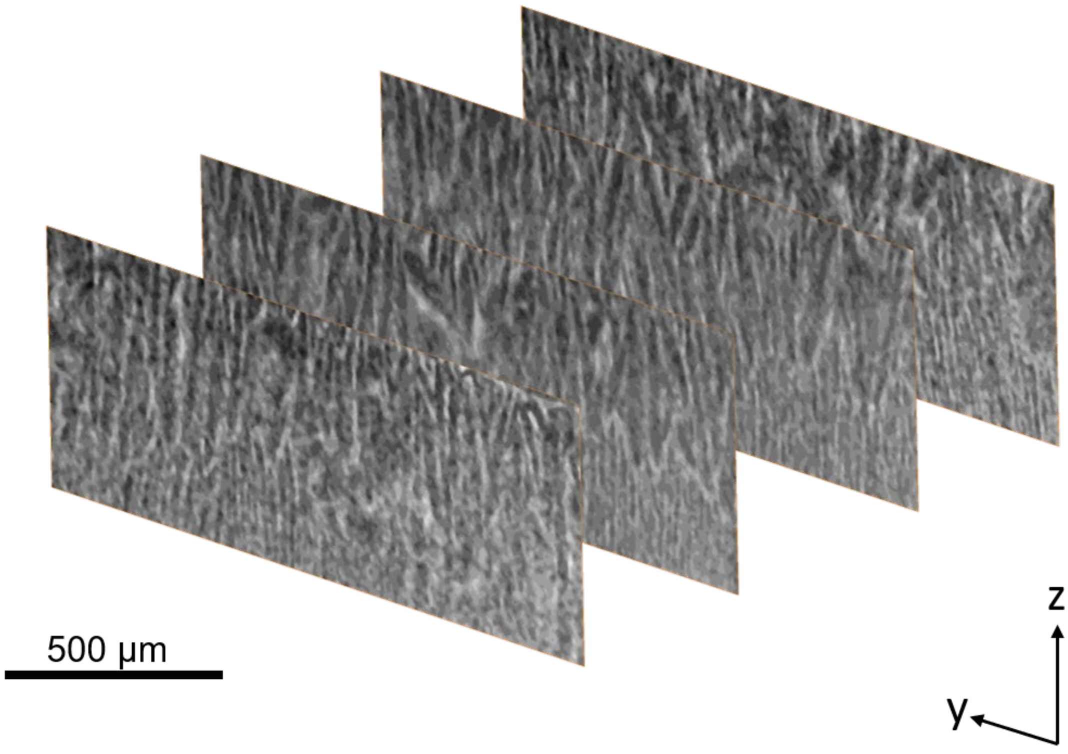

Fig. S10: Slices of 2D XCT images of the magnified DIT cathode along the *y*-*z* plane using a higher optical module.

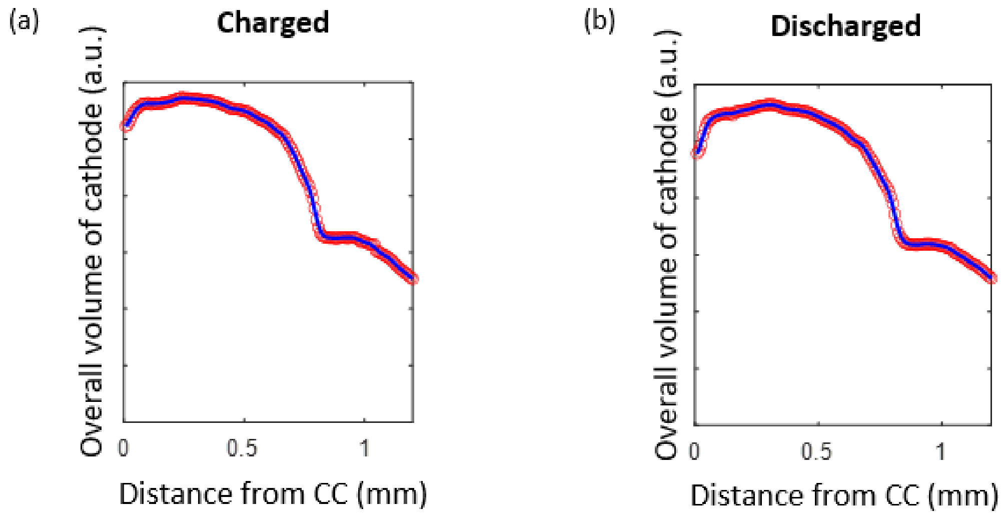

Fig. S11: Estimated DIT cathode volume against distance from the current collector at the charged and discharged states.

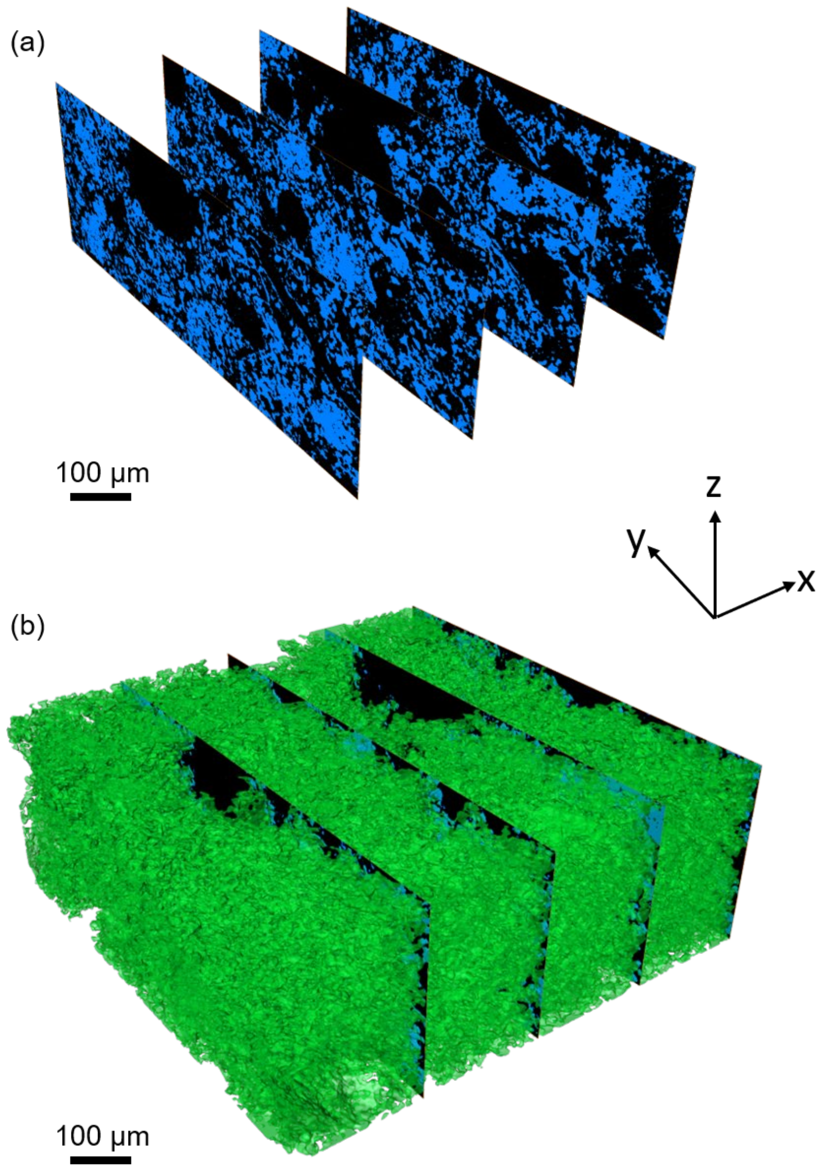

Fig. S12: XCT results of the SC electrode structure showing (a) 2D segmented slices along the *y*-*z* plane of the material (blue) and pore phases (transparent); and (b) zoom-in 3D reconstruction.

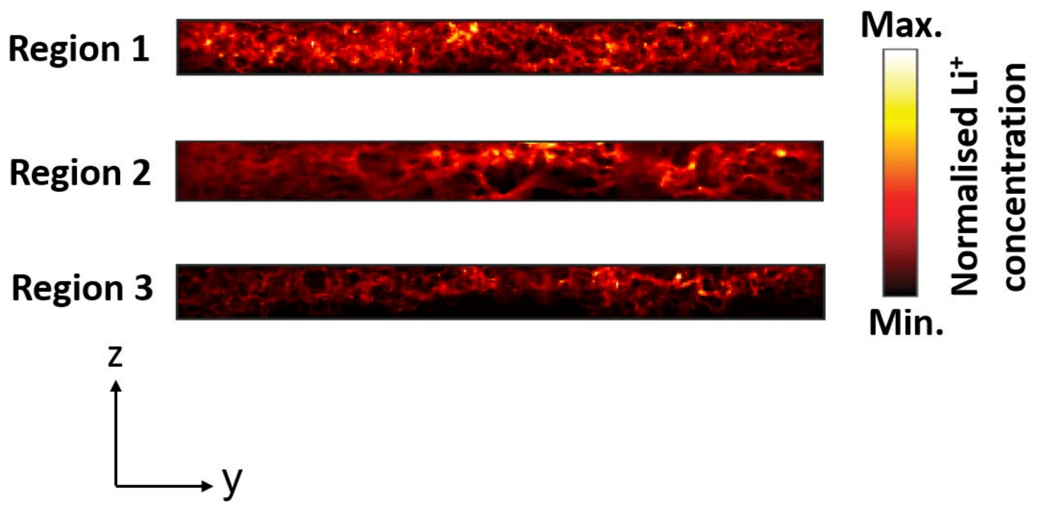

Fig. S13: Simulated Li^+ ion flux in the *y-z* direction in the three depth regions inside the SC electrode.

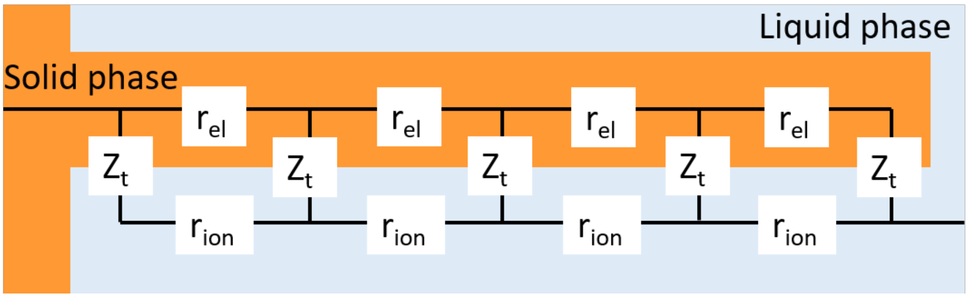

Fig. S14: Equivalent circuit model of the transmission line model (TLM) for fitting the results from simulated EIS along different directions and in different depth regions of the cathode.

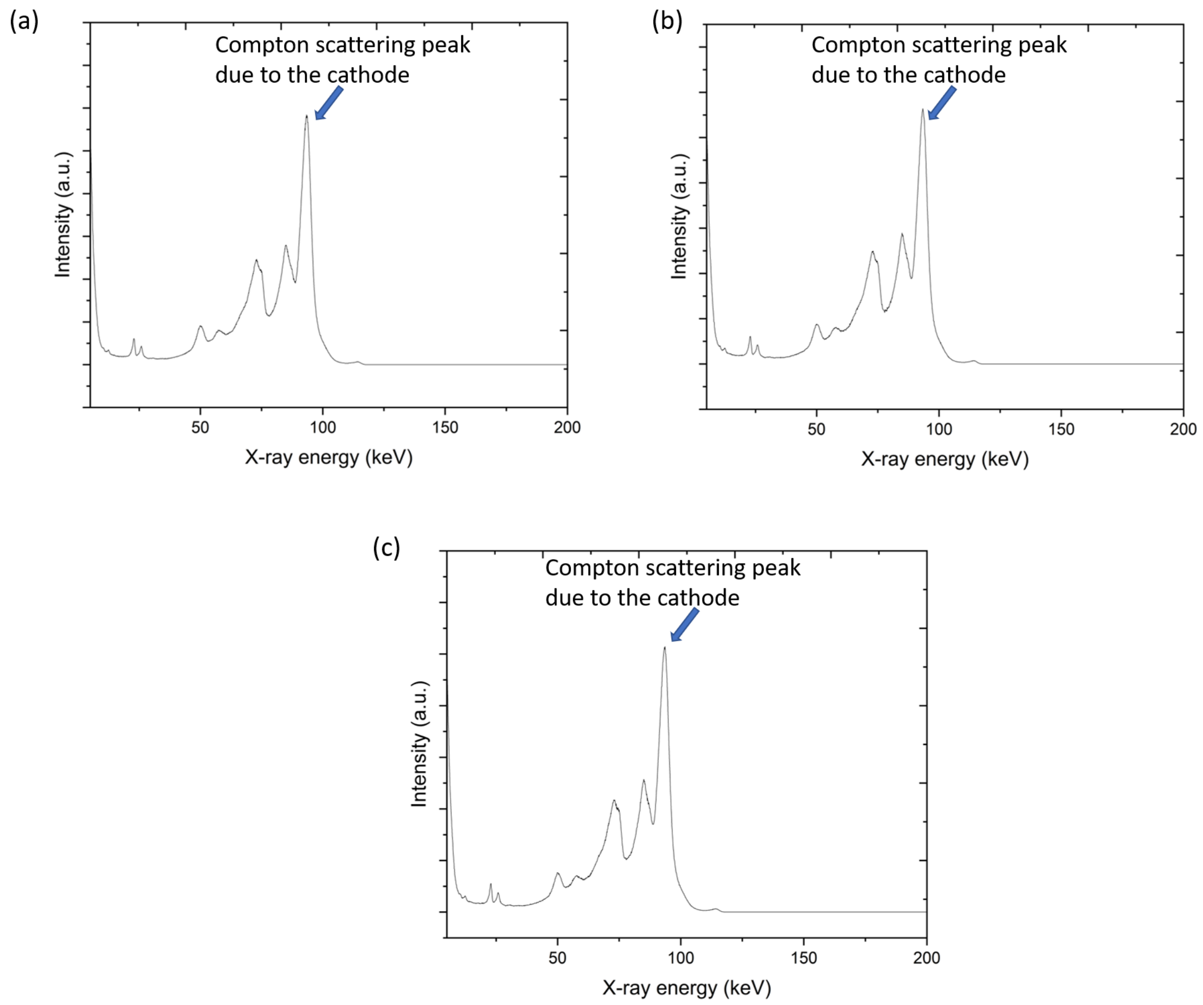

Fig. S15: Compton scattering X-ray energy spectra at 0-200 keV for the battery containing the NMC cathode at (a) region 1, (b) region 2, and (c) region 3 at the charged state.

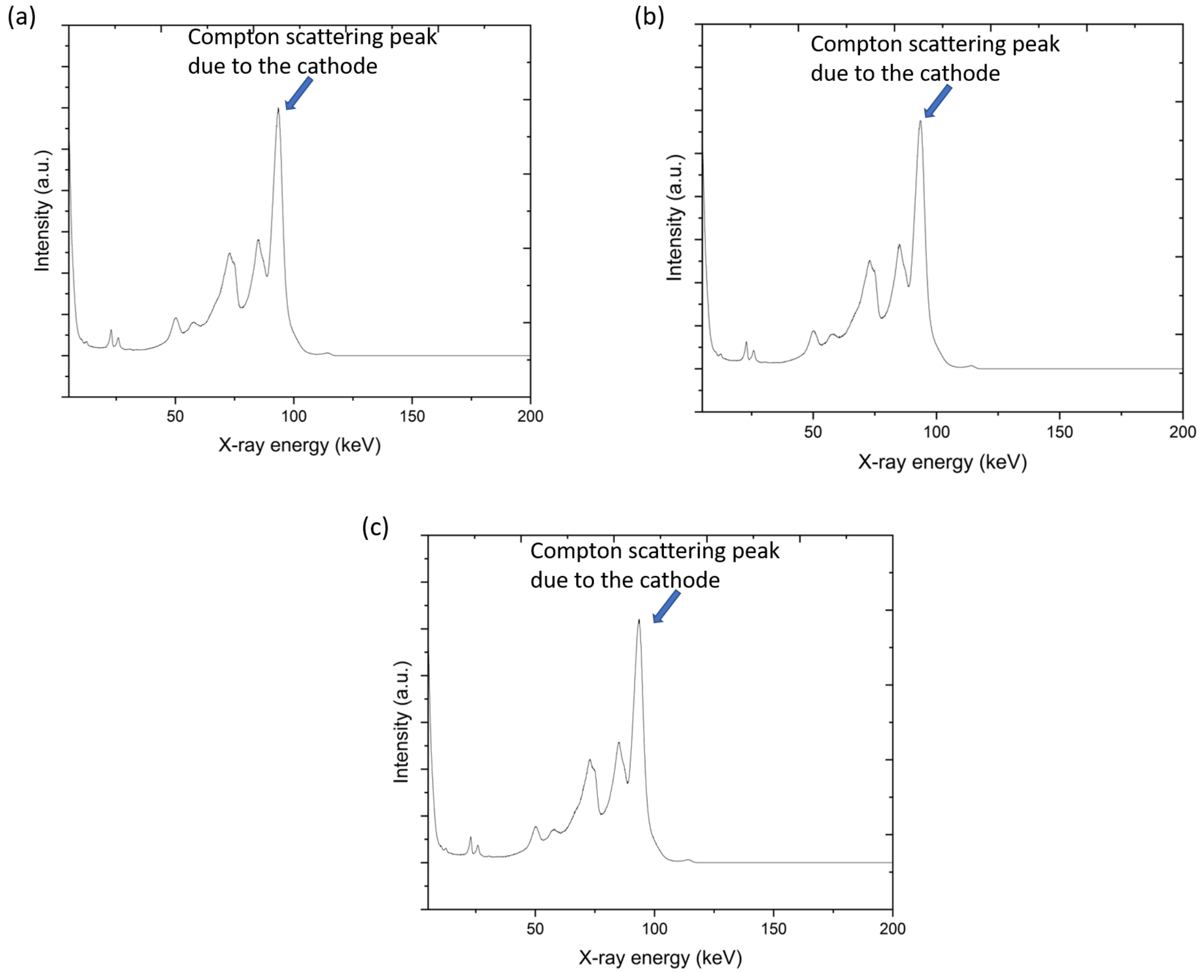

Fig. S16: Compton scattering X-ray energy spectra at 0-200 keV for the battery containing the NMC cathode at (a) region 1, (b) region 2, and (c) region 3 at the discharged state.

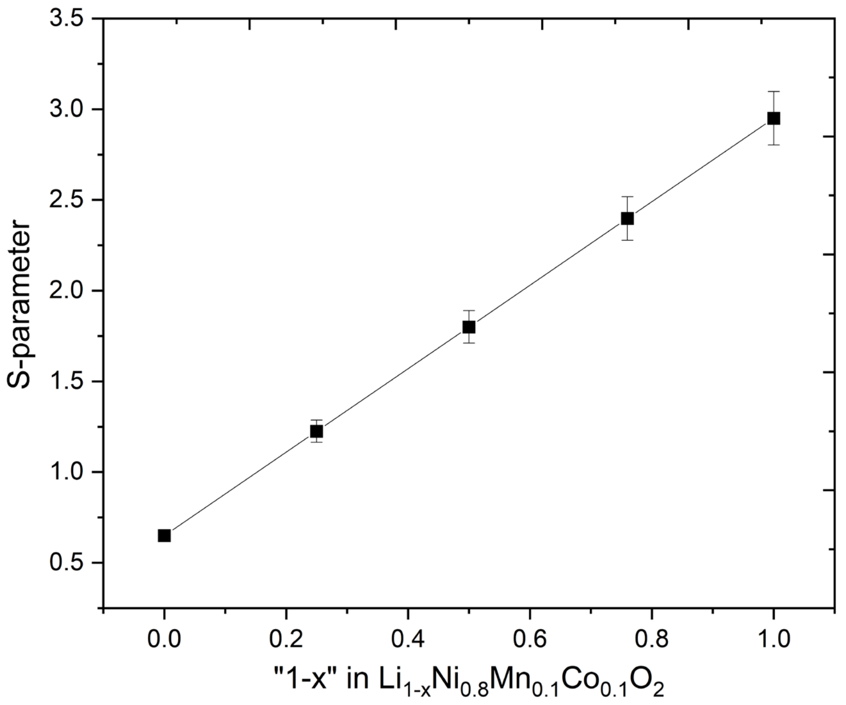

Fig. S17: A graph of “1-x” in $Li_{1-x}Ni_{0.8}Mn_{0.1}Co_{0.1}O_2$ against S-parameter after calibration with experimental results.

References:

[S1] Qin, G., Q. Ma, and C. Wang, A new route for synthesizing C/$LiFePO_4$/multi-walled carbon nanotube secondary particles for lithium ion batteries. Solid State Ionics, 2014. 257: p. 60-66.

[S2] Li, X., et al., Enhancement of electrochemical performances for $LiFePO_4$/C with 3D-grape-bunch structure and selection of suitable equivalent circuit for fitting EIS results. Journal of Power Sources, 2015. 291: p. 75-84.
